# Immunological aspects predicting metastatic sentinel lymph node in early breast cancer patients

**Published:** 2012-12-25

**Authors:** C Bordea, M Bordea, A Totan, I Condrea, S Voinea, A Sandru, M Plesca, A Blidaru

**Affiliations:** * Surgical Oncology Department, "Prof. Dr. Al. Trestioreanu" Institute of Oncology, Bucharest; ** ALCOS Medical Center, Bucharest; *** Biochemistry Department, “Carol Davila" University of Medicine and Pharmacy, Bucharest; **** Pathology Department, "Prof. Dr. Al. Trestioreanu" Institute of Oncology, Bucharest

**Keywords:** early breast cancer, sentinel lymph node, immunology

## Abstract

Tumour antigens are poorly expressed, heterogeneous and they modulate rapidly. As a result, their recognition and elimination by the immune system is very difficult. There are several mechanisms, by means of which, the host can neutralize oncogenesis and prevent it from occurring.

The sentinel lymph node concept has brought about a revolution in the surgical treatment of the regional lymphatic basin while preserving the prognostic value of the regional lymph node status in breast cancer.

This prospective study included 93 women with early breast cancer with initial indication for surgery in whom the sentinel lymph node technique was employed. Cell immune response was assessed prior to surgery by means of in vitro mononuclear cells blastic transformation assay (BLT), of immunoglobulin (Ig) and interleukin 2 (IL-2) measurements.

The results were correlated with tumour size, presence of positive sentinel lymph node, tumour proliferation and growth markers (Ki-67, c-erbB2, bcl-2).

Even in its less advanced stages, breast cancer is more aggressive and associates with an increased rate of sentinel lymph node metastases in patients below 50 years old, the tumour size exceeds 20 mm, with the presence of peritumoral lymphocytic infiltrate, positive Ki-67 and bcl-2, an alteration of T helper (Th) lymphocytes function, increased immune suppression through IL-2 decrease, signalled by blastic transformation indexes modifications and a drop in IL-2 production (p<0.01).

## Introduction

As a cause of death by malignant tumours in women, breast cancer ranks second after lung cancer as far as the number of cases handled by oncology centres throughout the country. Reducing mortality was possible as a result of the association of early detection of breast cancer with improved and diversified treatment methods [**1,2**].

The sentinel lymph node is the first lymph node that drains the lymph from the primary tumour [**3-5**]. This concept has brought about a revolution in the surgical treatment of the regional lymphatic basin in less advanced breast cancer and malignant melanoma [**6**]. With the introduction of sentinel lymph node identification and biopsy technique one could preserve the prognostic and aggressiveness assessment value of regional lymph node status while also reducing morbidities associated with complete lymphadenectomy.

The two important elements that are involved in the relationship between the tumour and the host’s immune system are the following: the tumour’s oncogenic and immunogenic potential and the existence of some immune system deficiencies. Immune control is based on the balance between tumour reducing mechanisms and the immune “stealth" facts.

Tumour antigens are poorly expressed, heterogeneous and they modulate rapidly. As a result, their recognition and elimination is very difficult. There are several mechanisms, by means of which, the host can neutralize oncogenesis and prevent it from occurring. Among such means one could mention cytotoxic T lymphocytes, macrophages having specific and non-specific cytotoxic functions, natural killer (NK) cells, various types of cells performing antibody-dependent cell-mediated cytotoxicity, antibodies, some lymphokines [**7,8**].

In breast cancer, just like in the case of other cancers, the progress of the disease depends on the summation of several aggressiveness factors. The purpose of the study was to assess and identify the tumour profile susceptible of higher aggressiveness in this process, by using immunological and pathological methods.

To that end, one has compared the immune status, pathological results both intraoperatively and in paraffin embedded tissue as well as the tumour immunohistochemistry of a group of female patients with early breast cancer, in whom axillary sentinel lymph node identification and biopsy technique has been performed [**9**].

## Methods and results

A radioactive tracer with Tc99 (0.5-1 mCi) was injected prior to surgery in the peritumoral area of 93 women with stage I and IIA breast cancer. Intraoperative detection of the sentinel lymph node was based on tracing the radioactive colloid that has migrated along a lymphatic route from the place of injection to the sentinel lymph node, the so-called “hot spot", by means of an intraoperative portable gamma probe [**10**]. Excision biopsy of the sentinel lymph node was performed and followed by an intraoperative histopathological examination and in paraffin-embedded sections. Surgical and oncological treatments performed afterwards have followed the present accepted guidelines in breast cancer.

 The parameters assessed in this study are listed below. (**Table 1**)

**Table 1:** Assessed parameters.

Clinical aspects	Immune status*	Pathology	Immunohistochemistry
Age	Lymphocytes blastic transformation (BLT)	Sentinel lymph nodes status	Estrogenic and progesterone receptors (ER and PR)
Hormonal status	Serum IgG, IgA, IgM	Tumour size and Histopathological	Her2 oncogene
Tumour diameter	IL-2	Peritumoral tissue invasion	Ki-67 proliferation markers
		Peritumoral lymphocytic infiltrate	bcl-2 protein

* The control group consisted of 20 apparently healthy women (blood donors). Results were expressed by calculating the mean value ± standard deviation (X ±DS). 

 Lymphocyte blastic transformation (BLT)

In order to test their blastic transformation ability in the presence of an antigen (mitogen: PHA – Phytohaemagglutinin, PWM – Pokeweed Mitogen, ConA – Concanavalin A), lymphocytes stimulation was performed in vitro, in mononuclear cell cultures isolated from the peripheral blood of the cases in the study. For every culture, 2 x 105 lymphocytes were collected in RPMI 1640 medium, to which 10% foetal serum (Gibco) was added. In every case, one non-stimulated control sample was necessarily processed and there were at least 3 tests (cultures) for each stimulated or non-stimulated sample.

Cultures were incubated for 48 hrs at 370C in 5% CO2 atmosphere. BLT degree measurement was performed by means of tritiated thymidine incorporated by the nucleus of the dividing lymphocytes. The count per minute (cpm) value for the control samples or the stimulated ones represented the mean of the three tests processed for each sample [**11**].

Assessment of cellular immune response by means of this test could be done either directly by comparing cpm absolute figures, or indirectly by calculating the BLT index, which represented the ratio of the cpm value in the sample with mitogen over the cpm in the control (non-stimulated) sample of the respective culture [**12**].

The values found in the healthy controls, expressed as blastic transformation index are listed below:

I. PHA 92.6 ± 8.4

I. PWM 35.7 ± 4.3

I. Con A 20.3 ± 2.8

I.PHA / I.Con.A (LTh /LTs) ratio 4.5

Measuring serum immunoglobulins: IgG, IgA, IgM

The turbidimetric test with spectrophotometric reading at 340 nm was used.

Reference (normal) values were as follows:

IgA = 70 - 250 UI/ml (1 – 3.6 g/l) – control group mean 2.1 ± 0.9 g/l

IgG = 100 - 200 UI/ml (8 - 16 g/l) - control group mean 13.9 ± 2.8g/l

IgM = 80 - 200 UI/ml (0.8 -1.7 g/l)- control group mean 1.6 ± 0.4g/l

Determining Interleukin 2 (IL-2) concentration in lymphocytes cultures

Interleukin 2 concentration was measured in the culture medium of PHA stimulated mononuclear cells after 24, 48 and respectively, 72 hours of culturing. An immunoenzymatic assay (ELISA) was used [**13,14**]. ELISA assays use antigens or antibodies attached to a solid phase (support) which is represented by a microtiter plate. If the complementary antibody or antigen is present in the sample, it will bind to its correspondent on the solid phase. The surplus will be removed by washing. In the presence of an enzyme conjugate and of a chromogenic substrate, a colorimetric reaction takes place, related to the Ag/Ac complex, which can be measured spectrophotometrically.

Primary tumour immunohistochemistry

A semi quantitative assessment was done by measuring the immunohistochemical reaction under optical microscope and counting the percentage of positive cells versus the total number of neoplastic cells [**15,16**]. The score varied from 0 to +++. (**Table 2**)

**Table 2:** Immunohistochemistry of the primary tumour

Immunohistochemistry	+	++	+++
ER	<20 % immunoreactivity	20 - 50 % immunoreactivity	>50 % immunoreactivity
PR	<20 % immunoreactivity	20 - 50 % immunoreactivity	>50 % immunoreactivity
Ki67	< 5%	5-30%	>30%
c-erb-B2	< 20 %	20-50%	>50%
bcl-2 was assessed as positive (+) or negative (-)

The group in the study included patients aged 31 to 69, the average age being 53.21. Two sub-groups in the studied group included a much larger number of cases: the 41 to 50 age group and the 51 to 60 age group that represented 29.03% (27 patients) and, respectively, 38.70% (36 patients) of all the patients in the study.

The average tumour diameter was of 28 mm (clinically), tumour size varying between 6 and 40 mm. Approximately half (44.08%) of the patients studied had tumours whose size varied between 21 and 30 mm.

The most frequently encountered histopathological type of primary tumour was invasive ductal carcinoma, which accounted for 76% of all cases.

The degree of tumour differentiation had the following distribution in the group under study: G1 – 25.81 % (24 patients), G2 – 34.4% (32 patients), G3 – 39.79% (37 patients).

Peritumoral tissue invasion was noted on histopathological examination in 47 cases (50,53%). A number of 60 tumours (64.51%) encountered lymphocytic peritumoral infiltrate on histopathological examination. 

Metastatic invasion of the sentinel lymph node was identified in 34 cases (36.55%). In 59 cases, the sentinel lymph node was clear of disease. 

In 64% of the patients (58 cases), estrogenic and progesterone receptors were positive. Tumour proliferation marker Ki-67 was found positive in 47 patients. c-erbB2 (Her-2/neu) oncogene was over-expressed in 24 cases (28.57%). bcl-2 was determined positive in 36 patients.

Immunologic assessments results

In the group of 93 patients with breast cancer we studied, a high dispersion of I.PHA values was noticed. Results led to groups split into two: one whose values were 3SD lower than the control group mean (55 cases), and the remaining cases (38) that made up the second group.

The mean values obtained in the 55 cases were:

I.PHA 54.4 ± 2.2

I.PWM 25.5 ± 3.4

I.Con A 23.1 ± 4.6

The I.PHA /I.Con A ratio (LTh / LTs) = 2.35

The mean values obtained in the 38 cases were:

I.PHA 77.2 ± 5.8

I.PWM 30.1 ±4.9

I.ConA 18.9 ± 3.1

The I.PHA / I.ConA ratio (LTh / LTs) = 4.08

In the group where IL-2 in the culture medium was measured after 48 hours from stimulation with PHA, in 40 cases the values were significantly lower (p<0.01) compared to the control group: 32 ±3.4 pg/0.1 ml compared to 44 ±4.1 pg/0.1 ml.

Serum immunoglobulin concentrations measured in the patients in the study group were the following:

Ig M (g/l) 2.4 ± 0.6

Ig G (g/l) 14.2 ± 3.2

Ig A (g/l) 2.2 ± 0.8.

 The mean value of IgM concentration in the studied group was higher than that of the control group (2.4 ± 0.6 compared to 1.6 ± 0.4g/l).

 T lymphocytes function is altered in the group of 55 patients with malignant tumours. This was made obvious by the drop in I.PHA as well as by a drop in IL-2 concentration in the mononuclear cultures from peripheral blood. I.ConA indicates a growth of suppression both through its absolute value but especially by the LTh (IPHA) and LTs (I.Con A) ratio.

Correlation between the lymphocytic peritumoral infiltrate and the sentinel lymph node status 

**Fig. 1:**
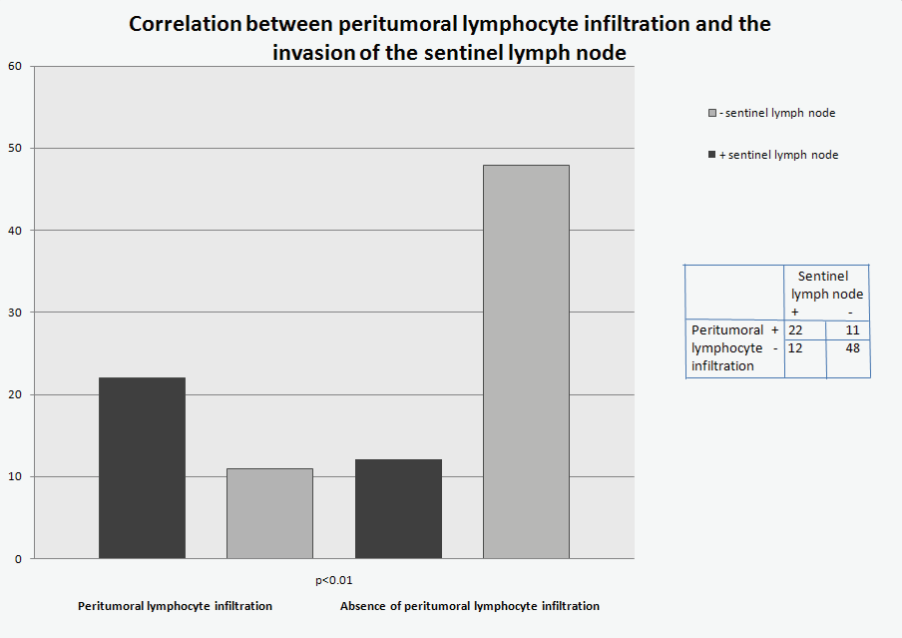
Correlation between peritumoral lymphocyte infiltration and sentinel node status

 A number of 22 tumours, where peritumoral lymphocytic infiltration was present, associated the presence of the neoplastic invasion in the sentinel lymph node (p<0.01). In 48 cases, where peritumoral lymphocytic infiltration was absent, the sentinel lymph node was free from disease (**Fig. 1**).

Immune response correlation depending on tumour dimension and axillary lymph nodes status

**Fig. 2:**
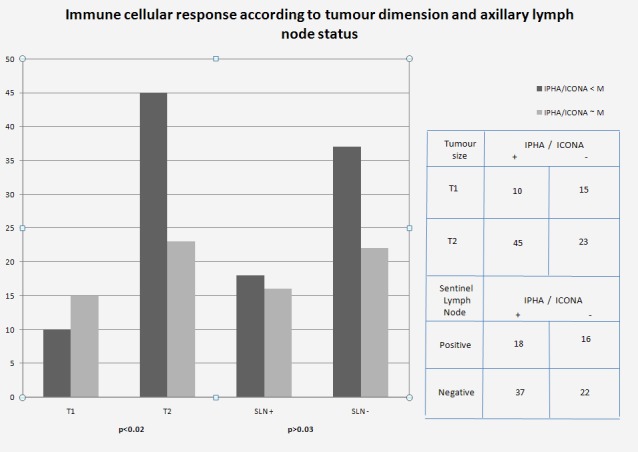
Cellular immune response according to tumour dimension and sentinel lymph node status

We noticed significant alterations of the cellular immune response, expressed by the change in the I.PHA/I.ConA ratio, in the patients whose tumours exceeded 2 cm in size (T2 in the TNM classification) (p< 0.02) (**Fig. 2**).

**Fig. 3:**
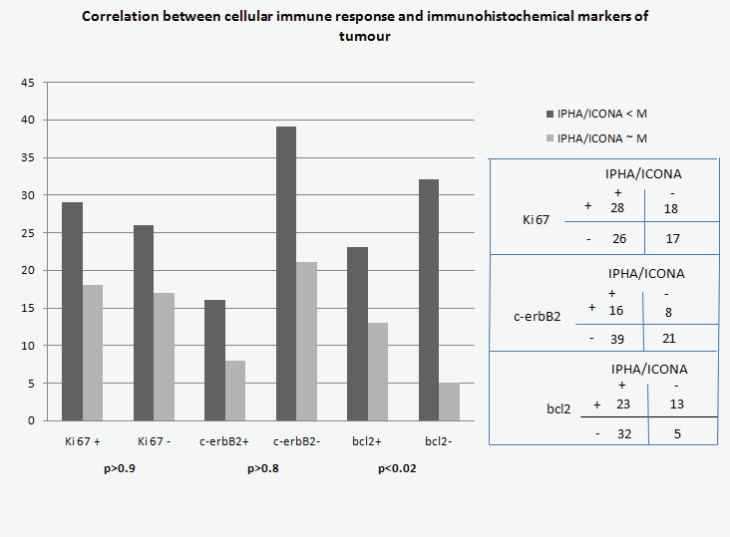
Cellular immune response according to tumour immunohistochemistry

Cellular immune response depending on tumour immunohistochemical markers Immune response was not influenced by the expression of tumour markers Ki-67 and c-erb B2. We identified a statistically significant reverse correlation with protein bcl 2 expression at a mitochondrial level (p<0.02). This may be due to the diminished apoptotic activity of tumour cells that express bcl 2 protein in a lower percentage (**Fig. 3**).

## Discussion

Peritumoral lymphocytic infiltrate is an important predictive factor for the metastatic invasion of the sentinel lymph node. This could help identify the cases more likely to be false negative [**17-18**]. In addition, peritumoral lymphocytic infiltrate significantly associates with the presence of lymph node metastases in invasive breast cancer [**19**]. In our study, a number of 22 tumours, in which peritumoral lymphocytic infiltration was present, associated with sentinel lymph node invasion (p<0.01).

Estrogenic receptors are positive in about 80% of women with breast cancer. In most of the cases, they associate with positive progesterone receptors [**20**]. Positive progesterone receptors, degree of tumour differentiation and tumour size are independent of prognostic factors for lymph node invasion in women with positive estrogen receptor breast cancer under the age of 50 [**21**]. Tumours with a high differentiation degree and without axillary metastases associate with a better survival rate. Breast cancer occurring in young women (under the age of 35) has a higher probability to have a low differentiation degree (G2, G3), to be ER(-), and to have a positive sentinel lymph node, thus expressing higher aggressiveness and a cautious prognostic [**22-24**]. In our study, G3 tumour differentiation degree associated more frequently with the metastatic invasion of the sentinel lymph node (in 21 cases), thus having a statistically significant value (p<0.01).

Tumour proliferation rate is a biological parameter that needs to be considered both in establishing the indication and in assessing the effectiveness of chemotherapy. Analysis of tumour proliferation markers depending on the sentinel lymph node status identified a direct correlation between Ki-67+ and sentinel lymph nodes with metastatic invasion (p<0,03). In the study group, Ki-67 expression directly correlates with the degree of histopathological differentiation of the tumour, the expression of this marker increasing to a statistically significant value in the less differentiated tumours (p<0.01).

C-erbB 2 is a prognostic independent factor for disease free and for overall survival as well as for the presence of axillary lymph node metastases. Tumours expressing c-erbB 2 have an aggressive behaviour. Evaluation of this latter parameter in patients with breast cancer can provide a better treatment plan and a better prognostic outcome [**25,26**]. Patients with negative sentinel lymph nodes and with c-erb B 2 overexpression have a cautious prognostic when this associates with tumours larger than 2 cm, G3 and lymphovascular invasion [**27**]. In the studied group, c-erb B 2 over expressed in the primary tumour did not associate to a statistically significant degree with the presence of sentinel node metastases (p>0.3).

Cellular control of immune response is primarily performed by means of T suppressor lymphocytes. Among the factors favouring the proliferation of the LTs function are also the following: low antigen doses, long antigen persistence and inability of macrophages to capture or process antigens [**28**]. The reverse correlation between lymphocytic function alteration and bcl 2 protein expression in the cases we studied, confirms the poor apoptotic activity of tumour cells (p<0.02). Our results show that, in most of the cases, LTh functionality is impaired (I.PHA lower than in the control group).

Increased immunosuppression has been frequently associated with the onset and progress of cancer. In our study, I. Con A, but more particularly the ratio between I.PHA and I.Con A (LTh/LTs) change thus favouring increased immunosuppression in 55 patients with altered immune response - 2.35 versus 4.08 in 38 patients and, respectively, 4.5 in the control group. Our study points to a direct, statistically significant correlation between cellular immune response and tumour size: tumours larger than 2 cm in size associate with alteration of the lymphocytic function (p<0.02).

Data in literature show that immune reactivity of the regional lymph node is affected by the immunosuppressive factors released by the primary tumour and drained through the lymphatic system. T suppressor lymphocytes activity is higher in the tissues closer to the tumour, while lymphocytes in the regional lymph nodes produce a poorer response on stimulation with mitogens [**29-30**]. In the study group, the cases with a positive sentinel lymph node were accompanied by the alteration of Th lymphocytes function. LTh failure, either primary or as a result of increased immunosuppression, resulted in a drop of IL-2 quantity in the mononuclear cells culture from peripheral blood. (p<0.01).

Poor lymphocytic stimulation through IL-2 may be due not only to the changes in IL-2 concentration but also to T cells receptors alteration. Peripheral blood contains LT precursors that have the ability to recognize tumour antigens and that send out an activation signal to the LTh cells and to the cytotoxic T lymphocytes (LTc) via cytokines IL-2 [**31**]. Lymphocyte activation translates in IL-2R receptor expression on the cell surface. Therefore, IL-2 plays a special role in activating killer cells. Failure to produce or recognize IL-2, results in impaired tumour lyses. In vitro incubation of lymphocytes with IL-2 increased the cytotoxic ability directed against tumour cells. The addition of anti IL-2 antibodies in the stimulated cultures had as an effect the disruption of immunocompetent cells proliferation.

Mean value of IgM concentration was higher in breast cancer patients compared to controls (2.4 g/l versus 1.6 g/l). In breast cancer, IgM-type antibodies may be produced, but not in all histopathological forms. Humoural immune response seems to be disrupted at the level of the primary response (IgM production) and is not followed by the production of IgG-type antibodies that could consolidate and complete humoural immune response. IgM increase in the incipient stages of breast cancer could contribute to inhibiting immune control performed by NK. That is why in this pathology, humoural immunity is not an efficient control and removal mechanism of neoplastic antigens [**32**].

Moreover, the aggressiveness factors of the disease are compounded by the impact of surgery and anaesthesia, which, by increasing the level of catecholamines, of glucocorticod hormones, result in a decrease of the LTc/LTs ratio, of NK activity, in the weakening of the natural barriers of collagen structures, the inhibition of the activity of the complement system, the impairment of the activity of macrophages, decrease in the IL 1, IL 2 production, decrease in the number of lymphocytes and impairment of their functionality [**13-32**].

Alessandro Santin commented upon the role of regional lymphadenectomy in the light of observations referring to experiments of cytotoxic T cells activation [**33**]. This work suggests that cytotoxic T cells are activated by the recognition of tumour antigens only after tumour cells are at least partially degraded in the draining lymph nodes. Antigenic tumours become immunogenic enough in order to activate antigen-specific native T cells only during the interaction between the T cells and the tumour cells in an environment that enhances antitumor reaction, namely the lymph nodes. Although the activation is rarely complete, this analysis raises questions about the treatment plan that includes the removal of immunocompetent lymph nodes together with the lymph nodes with metastatic disease and immune suppression triggered by the tumour, when the sentinel lymph node technique is not employed.

## Conclusions

The immune response deficiencies in patients with stages I and IIA breast cancer, with primary surgical treatment, highlighted in this study are: impaired functionality of Th lymphocytes and increased immunosuppression, evidenced by the drop in IL-2 production (p<0.01); reverse correlation between lymphocytic function alteration and bcl-2 protein expression confirm the poor apoptotic activity of tumour cells (p<0,02); alteration of the lymphocytic function supported by the change in the I.PHA/I.ConA ratio associates with a tumour diameter greater than 2 cm (p<0.02). These breast cancers are more aggressive and are associated with an increase frequency of sentinel lymph node metastases. 

 The fact that these immunologic changes occur in the less advanced stages of the disease suggests the idea that malignity is an effect of immunological deficiencies, too
